# Spleen-Preserving Surgery in Splenic Artery Aneurysm

**DOI:** 10.1155/2017/8716962

**Published:** 2017-12-17

**Authors:** Ulaş Aday, Emre Bozdağ, Ebubekir Gündeş, Selçuk Gülmez, Kamuran Cumhur Değer

**Affiliations:** Department of Gastroenterological Surgery, Kartal Koşuyolu High Speciality and Training Hospital, Kartal, Istanbul, Turkey

## Abstract

Endovascular interventions are increasingly used in the treatment of a splenic artery aneurysm (SAA), which is a rare and life-threatening clinical disorder. However, in cases of SAA rupture, minimally invasive interventions are unsuitable, and open surgery remains the gold standard method. In open surgery, care should be taken to preserve the spleen and its immune function in cases where an arterial segment of sufficient length allows for reconstruction. An SAA was detected in a 51-year-old woman who presented to our polyclinic with left upper quadrant pain. An endovascular intervention was unsuccessful, and open surgery was performed. Approximately 5 cm of aneurysm in the middle segment of the splenic artery was treated by arterial anastomosis, and the spleen was preserved. The patient experienced no postoperative complications and remained asymptomatic at the seventh month of follow-up. The aim of this case report is to emphasize the importance of splenic sparing surgery in cases of SAAs.

## 1. Introduction

A splenic artery aneurysm (SAA) is a rare condition, in which the diameter of the splenic artery dilates to over 1 cm. SAA rupture is associated with relatively high mortality [1]. The risk of rupture is increased in cases of pregnancy, pseudoaneurysms, aneurysms with a diameter greater than 2 cm, portal hypertension, symptomatic SAAs, and liver transplantations. In the presence of the aforementioned factors, treatment should take place without delay [1, 2]. There is insufficient evidence on the best treatment for SAAs due to the retrospective nature of literature studies and low number of cases [2]. With advances in medicine, endovascular treatment (coil embolization or stenting) and laparoscopic surgery are increasingly used to treat SAAs. However, open surgery remains the gold standard and most frequently applied treatment [3]. In open surgery, the aneurysm is frequently resected with splenectomy. Due to the important immune function of the spleen, spleen-preserving surgery is recommended whenever possible [4].

In this study, arterial reconstruction was performed in a symptomatic SAA patient to preserve the spleen.

## 2. Case Report

**Figure 1:**
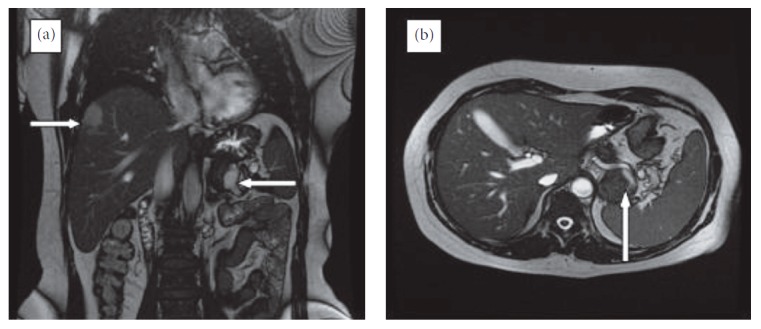
Magnetic resonance imaging of the hepatic hemangioma ((a) upper right arrow) and splenic artery aneurysm.

**Figure 2:**
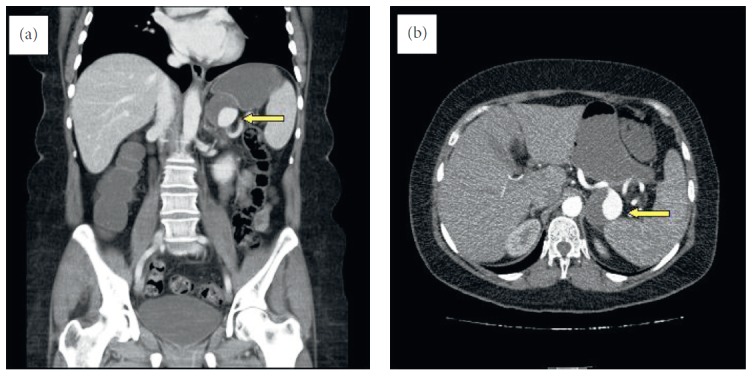
Multislice computed tomographic image of the splenic artery aneurysm.

A 52-year-old female presented to our polyclinic with increasing pain in the left upper quadrant in the previous month. She had been treated for hypertension and had a hysterectomy 7 years ago due to a myoma. The patient had quit smoking 3 years earlier and was taking alprazolam 0.5 mg/day for an anxiety disorder. A physical examination revealed minimal sensitivity of the left upper quadrant upon deep palpation and an incision scar from the gynaecologic surgery. Nothing else of note was detected. The patient's body mass index was 32.5 kg/m^2^, and laboratory measurements were within the normal range. Endoscopy and a colonoscopy conducted to determine the cause of pain revealed no pathological findings. An ultrasonic evaluation revealed no evidence of an aneurysm. However, magnetic resonance imaging (MRI) was conducted due to the presence of a hyperechoic lesion (53 × 39 mm in size) in the right lobe of the subcapsular area of the liver. The MRI study revealed a 35 mm diameter hemangioma in the liver segment 8 and a 40 mm aneurysm with a thrombus in the splenic artery of the pancreas tail region (Figure 1). Tortuosity of the splenic artery, which had a proximal diameter of 1 cm, was detected. In angiographic multislice computed tomography (CT), minimal aneurysmatic dilatation was detected in the celiac truncus in the midsegment of the splenic artery, in addition to saccular aneurysmal dilatation, 50% thrombosed and in the size of 45 × 40 × 39 mm adjacent to the pancreas. A long segment of the splenic artery transection extended from the distal aneurysm to the splenic hilus (Figure 2).

**Figure 3:**
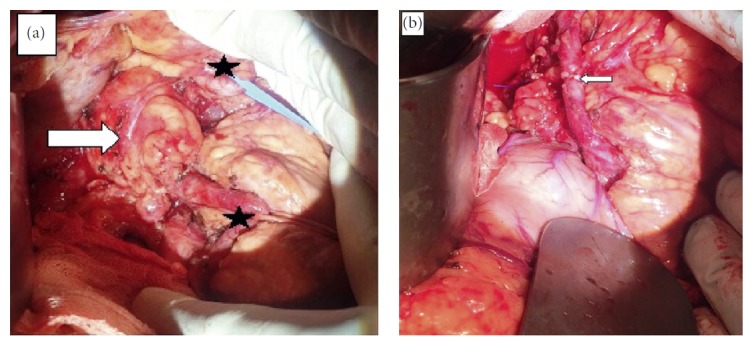
Intraoperative image of an aneurysm (a). The proximal and distal ends of the splenic artery have been marked with an asterisk. Splenic artery after anastomosis (b).

**Figure 4:**
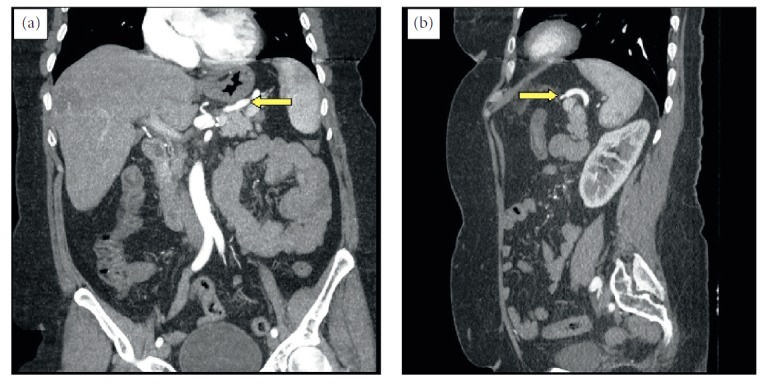
Computed tomographic image taken six months after surgery.

Following a consultation with the interventional cardiovascular department, an endovascular intervention was planned for the treatment of the aneurysm. Endovascular interventions made from the femoral region were inconclusive because of extreme angulation of the truncus celiacus, aneurysmal dilatation of the trunk, and tortuosity of the splenic artery. The patient was informed about the surgical procedure, and written consent was obtained. *H. influenza* and pneumococcal vaccinations were performed. The gastrocolic ligament was opened by median laparotomy, and the aneurysm was revealed. In the middle segment of the splenic artery, approximately 5 cm of aneurysm, which was adherent to the upper pancreas and retroperitoneal area, was opened by controlling the proximal and distal flow of the splenic artery. Collateral flow was not monitored. The proximal and distal ends of the splenic artery were anastomosed to provide flow continuity (Figure 3). The posterior and inferior wall of the aneurysm could not be resected because of excessive adhesions. The duration of the surgery was 185 min, and blood loss measured intraoperatively was 200 ml. No complications were observed in the postoperative follow-up period, and the patient was discharged without any problems on the fourth day. The patient was asymptomatic at the sixth month of postoperative follow-up. In angiographic CT, splenic artery flow was normal, and the residual aneurysm had regressed (Figure 4).

## 3. Discussion

Visceral artery aneurysms are rare, and most are SAAs. They are most commonly seen in patients in their 50s and 60s and show a female predominance [1, 5]. The aneurysm is thought to form as a result of degeneration of the arterial wall and deterioration of its elastic structure. Atherosclerosis, autoimmune pathologies, hypertension, smoking, pancreatitis, portal hypertension, trauma, and collagen tissue diseases have been associated with the formation of aneurysms [1, 4, 6]. Eighty percent of SAA cases are asymptomatic and incidentally detected in radiological evaluations performed for different clinical conditions. Pain in the epigastric and left upper quadrant is the most common symptom [3]. Seventy-five percent of ruptured aneurysms occur in pregnancies, and the mortality rate in cases of ruptured aneurysms is relatively high. The risk of rupture is higher in cases of pregnancy, pseudoaneurysms, aneurysms with a diameter greater than 2 cm, portal hypertension, and liver transplantations [1–4].

Although open surgery is accepted as the gold standard for SAAs, debate continues among clinicians about the ideal treatment. Perioperative morbidity and mortality rates are higher [7]. Recently, transcatheter embolization and endovascular stents have been increasingly used to treat SAAs. Given their high success rates and minimal invasiveness, these appear to be valid alternatives to open surgery. Laparoscopic surgery is widely used. However, it requires advanced skills and experience in vascular reconstruction [1, 6, 7]. In a meta-analysis, Hogendoorn et al. [2] found no significant difference between open surgery and endovascular repair in terms of major complication rates. Moreover, in this meta-analysis, the short-term outcome of endovascular surgery was better than that in open surgery, but open surgery was superior in terms of long-term outcomes [2]. Excessive angulation of the celiac axis and tortuosity of the splenic artery may result in failure of endovascular procedures. Open surgery should be the preferred choice for ruptured aneurysms. A splenectomy is frequently performed in conventional surgery, and aneurysm resection is then conducted if there is no excess adhesiveness. In cases of aneurysms that are sufficiently far away from the spleen hilum, the preservation of the spleen and its immune function should be a priority [1]. Open surgery using an anterior approach, a short gastric artery, and left gastroepiploic artery attachment increase the risk of splenic infarction. In such cases, a lateral retroperitoneal approach is recommended for the protection of collaterals [4, 8]. For giant SAAs or cases where a simple aneurysmectomy is impossible due to dense strictures, the preferred treatment options include the following: an aneurysmectomy plus splenectomy; bipolar splenic artery ligation, with or without an aneurysmectomy; transaneurysmal splenic artery ligation; or distal pancreatectomy, if necessary [9]. In our case, an endovascular intervention was first planned, but open surgery was subsequently performed due to the failure in cannulation of the celiac truncus, tortuosity in the splenic artery, and accompanying celiac aneurysm. The presence of an arterial segment of suitable length in the spleen allows for reconstruction and splenic sparing surgery. In the present case, an anterior approach was preferred because the need for revascularization was predicted.

Rupture of an SAA is a rare and life-threatening disorder. In symptomatic young women planning future pregnancies, treatment should take place without delay in cases of 2 cm deep aneurysms. Minimally invasive procedures should be the first choice in the treatment of SAAs. In appropriate cases, preservation of the spleen and splenic artery reconstruction are possible in open surgery.

## References

[1] Uy P. P. D., Francisco D. M., Trivedi A., O’Loughlin M., Wu G. Y. (2017). Vaskuler disease of the spleen: a review. *Journal of Clinical and Translational Hepatology*.

[2] Hogendoorn W., Lavida A., Hunink M. G. (2014). Open repair, endovasculer repair, and conservative management of true splenic artery aneurysms. *Journal of Vascular Surgery*.

[3] Akbulut S., Otan E. (2015). Management of giant splenic artery aneurysms: comprehensive literature review. *Medicine*.

[4] Al-Habbal Y., Christophi C., Muralidharan V. (2010). Aneurysms of the splenic artery-a review. *Surgeon*.

[5] Uyar I. S., Okur F. F., Akpinar B. (2013). A giant splenic artery aneurysm: a case report. *Turkish Journal of Thoracic and Cardiovascular Surgery*.

[6] Lakin R. O., Bena J. F., Sarac T. P. (2011). The contemporary management of splenic artery aneurysms. *Journal of Vascular Surgery*.

[7] Xin J., Xiao-ping L., Wei G. (2011). The endovascular management of splenic artery aneurysms and pseudoaneurysms. *Vascular*.

[8] Moore S. W., Guida P. M., Schumacher H. W. (1970). Splenic artery aneurysm. *Bulletin de la Societe Internationale de Chirurgie*.

[9] Yagmur Y., Akbulut S., Gumus S., Demircan F. (2015). Giant splenic artery pseudoaneurysm: a case report and literature review. *International Surgery*.

